# Residual Barriers for Utilization of Maternal and Child Health Services: Community Perceptions From Rural Pakistan

**DOI:** 10.5539/gjhs.v8n7p47

**Published:** 2015-11-03

**Authors:** Zahid Memon, Shehla Zaidi, Atif Riaz

**Affiliations:** 1 Department of Community Health Sciences, Aga Khan University Karachi, Pakistan; 2 Women and Child Health Division, Aga Khan University Karachi, Pakistan

**Keywords:** community barriers, child health, maternal health, Pakistan, rural areas

## Abstract

Low utilization of maternal and child care services in rural areas has constrained Pakistan from meeting targets of Millennium Development Goals (MDGs) 4 and 5. This study explores community barriers in accessing Maternal and Child Health (MCH) services in ten remote rural districts of Pakistan. It further presents how the barriers differ across a range of MCH services, and also whether the presence of Community Health Workers (CHWs) reduces client barriers. Qualitative methods were used involving altogether sixty focus group discussions with mothers, their spouses and community health workers. Low awareness, formidable distances, expense, and poorly functional services were the main barriers reported, while cultural and religious restrictions were lesser reported. For preventive services including antenatal care (ANC), facility deliveries, postnatal care (PNC), childhood immunization and family planning, the main barrier was low awareness. Conversely, formidable distances and poorly functional services were the main reported constraints in the event of maternal complications and acute child illnesses. The study also found that clients residing in areas served by CHWs had better awareness only of ANC and family planning, while other MCH services were overlooked by the health worker program. The paper highlights that traditional policy emphasis on health facility infrastructure expansion is not likely to address poor utilization rates in remote rural areas. Preventive MCH services require concerted attention to building community awareness, task shifting from facility to community for services provision, and re-energization of CHW program. For maternal and child emergencies there is strong community demand to utilize health facilities, but this will require catalytic support for transport networks and functional health care centers.

## 1. Background

Inadequate utilization of maternal and child health (MCH) services remains a major challenge across a number of low and middle income countries. Due to low utilization of MCH services, progress towards the Millennium Development Goals (MDGs) has been stalled in a subset of countries such as Pakistan (Borghi, Ensor, Somanathan, Lissner, & Mills, 2006; Shadoul, Akhtar, & Bile, 2010; Bryce, Black, & Victora, 2013). Over the last fifteen years, innovations in health systems such as pay for performance (Basinga et al., 2011), contracting (Danel & La Forgia, 2005), cash transfers and voucher schemes (Lim et al., 2010); have attempted to improve MCH services in many developing countries. While several countries have benefitted from rapid service coverage increase, a subset of countries has only seen a slow and incremental gain.

While there are numerous studies quantitatively identifying determinants of MCH services utilization, the volume of in-depth qualitative evidence is less. Further attention is needed in the poorly performing countries to probe and address residual barriers faced by clients so as to shape relevant health system interventions. There has been a steady expansion in barrier studies in the area of maternal services, particularly over the last decade. These studies have identified the most commonly cited barriers as: traditional and familial influences on decision of place for child birth; mistreatment and misbehavior by health workers; and perceived high cost of facility based childbirth (Afsana & Rashid, 2001; Amooti-Kaguna & Nuwaha, 2000; Chi, Bulage, Urdal, & Sundby, 2015; Mwangome, Holding, Songola, & Bomu, 2012). Barriers to child healthcare service utilization have been less well explored, however issues of mistrust of public facilities, and fear of adverse effects in case of vaccination have been commonly reported (Ganle, Parker, Fitzpatrick, & Otupiri, 2014; Mills, Jadad, Ross, & Wilson, 2005).

In order to unravel residual barriers three areas should be examined. Firstly, there has been less work undertaken on the comparative analysis across the range of MCH services to identify similar or differing barriers across services. Secondly, barrier studies currently provide the perception of mainstream clients. More contextual evidence is needed from disadvantaged population groups that fail to benefit from the service increase trends throughout the rest of the country. Thirdly, while there has been a spurt in outreach CHW programs in many countries, there is need to explore whether such programs have reduced the community perceived barriers to care seeking compared to other less well-resourced areas.

We present here findings from some of the most underdeveloped rural districts of Pakistan on: i) client related barriers reported by pregnant women and their spouses in seeking MCH care; ii) how the barriers differ across emergency, routine curative and preventive care services; and iii) whether there are any differences in perceived barriers across CHWs covered and non-covered areas.

## 2. Setting

Pakistan is the sixth most populous country in the world (United Nations, 2015), and Sindh province with its estimated population of 43 million, exceeds the population of many countries. Under five mortality in Pakistan is 89/1000 live births and maternal mortality ratio is 276/100000 live births (Pakistan Demographic and Health Survey, 2007 & 2013) falling short of expected MDG targets. In Pakistan, increased service utilization in more difficult to reach areas is required to fill residual gaps in service coverage. Several rural districts remain socio- economically underdeveloped, are located quite far from services, with poorly functional government health services, and negligible private health sector presence (Technical Resource Facility, 2011).

In Pakistan, only 38.8% of births were attended by skilled providers, while figures for rural areas are 29.8% (PDHS, 2007). Similalry, only 21.7% of married women of reproductive age in Pakistan were using any modern method of contraception, with the figure for women in rural areas even lower i.e. 17.7%. Although the difference is narrower than for maternal care, childhood immunization rates also vary between overall country figures (47.3%) and the figure for rural Pakistan (44.0%) (PDHS, 2007).

Traditionally there has been a policy emphasis in Pakistan on expansion of health facility infrastructure through successive annual development plans. Primary healthcare services are delivered via an extensive network of 5,336 Basic Health Units (BHUs) and 560 Rural Health Centres (RHCs) (Ministry of Finance, 2006-07). Contracting out of Basic Health Units to another government funded entity was initiated in 2008 with the rationale of improving health facility infrastructure and functionality.

Although Pakistan has a number of outreach programs, however these have suffered from poor governance and insufficient resource allocation (Karim, Zaidi, & Mahmood, 1999). These include the flagship community health worker outreach program, a special vertical program for Maternal, Neonatal and Child Health (MNCH), and an expanded program on immunization (EPI). EPI was launched in 1978 and is providing static and outreach services in Pakistan to protect children against vaccine preventable diseases. EPI currently vaccinates children against tuberculosis, polio, diphtheria, pertussis, tetanus, hepatitis B, haemophilus influenza type B, and pneumococcal diseases. The Lady Health workers (LHWs) program was launched in 1994 to supply contraceptives at the doorstep and provide health education for MCH and EPI services. However, only 45% of areas are covered by LHWs because entry level qualifications result in few recruits (Oxford Policy Management, 2009). The national MNCH program started in 2007 and operates in most districts of Pakistan, aiming to improve skilled delivery through placement of community based midwives.

This study focuses on ten of the most underdeveloped rural districts of Sindh province identified through periodic multiple cluster surveys undertaken by UN agencies, the MNCH program and Health Department of Government of Sindh. These districts were earmarked for placement of result based funding innovations through the Norwegian-Pakistan-Partnership Initiative (NPPI) to improve the MCH coverage rates across the province. The districts included Badin, Jamshoro, Tharparkar, Umerkot, Nawabshah, Larkana, Shikarpur, Kamber, Kashmore and Ghotki. The NPPI initiative in these ten districts was implemented through the MNCH program with the purpose of identifying the measures needed to improve MCH access. This study is part of the baseline studies conducted in 2008-09 for the NPPI initiative to assist in identification and design of necessary innovations. Table 1 provides socio-demographic information of the selected districts.

**Table 1:** Socio-Demographic Status of selected districts

Districts	Population^1^	% Literacy^1^	Household size^1^	Poverty Rank^2^ (1-16)	TFR^1^
**Badin**	1,355,564	32	7.2	6	6.01
**^φ^Umarkot**	945,641	37	7.0	11	6.58
**Shikarpur**	1,013,510	37	7.7	1	5.83
**Ghotki**	1,333,962	40	6.9	4	6.38
**^φ^Jamshoro**	776,265	42	7.4	5	6.83
**Tharparkar**	1,251,455	36	6.6	10	6.41
**Larkana**	1,321,701	37	7.8	3	6.18
**^φ^Qambar**	1,218,228	37	7.8	3	6.18
**^φ^Kashmore**	641,056	28	6.7	7	5.72
**Nawabshah**	1,288,733	37	7.2	9	6.14

φ Data for newly created districts of Umarkot, Jamshoro, Qambar and Kashmore was taken from parent districts and actual figures are expected to be lower.

1 *Source:* Multiple indicator cluster survey (MICS) 2003-04;

2 Social policy and development centre (SPDC) 2007.

## 3. Methodology

This study used qualitative exploratory design and explored behavioral dynamics, health seeking pattern and barriers for utilizing MCH services. Focus group discussions (FGDs) were conducted with purposively sampled research participants. The target population included i) mothers of children under five, ii) fathers of children under five and iii) LHWs as community level key informants. Altogether 60 FGDs were conducted, 20 with each of the target populations, involving 10 participants per FGD on average. Topic guide was developed for FGDs using literature review and modified according to the local context. It was finalized after consultation with the representatives of UN agencies, provincial government, and MNCH program. FGD guide was pre-tested in one district before initiation of data collection. The topic guide was developed around the themes of ANC, maternal complication, delivery, family planning, immunization, acute childhood illness. Box 1 provides main topics explored in FGDs.

Box 1 **Topics explored for each type of MCH service:**

- Utilization of service
- Preference of provider for utilization and reasons
- Barriers to utilization of MCH services

The catchment of public health care facility within each *Taluka* (sub-district) of the selected district was defined as a cluster for this study. Two clusters from each study district were selected randomly to collect data. Hence, 20 clusters were sampled across 10 districts.

Mothers and fathers of children under five were invited for voluntary and informed participation in the FGDs. One set of client FGDs was conducted in the villages close to the first level care facility (FLCF) within the distance of five kilometers (LHWs covered areas) and the other set of the FGDs was conducted in the peripheral villages of FLCF at a distance of 10-15 kilometers (LHWs un-covered areas) in each district. The villages were selected in consultation with the staff of the selected health facilities. FGDs with LHWs involved random selection of 10 LHWs from the list of LHWs available with each selected health facility. Altogether 1 FGD of LHWs, 1 of mothers and 1 of fathers was held per health facility catchment. Informed consent was taken from all study participants and confidentiality was maintained by entering data against anonymous codes.

Using a semi-structured guide, a trained moderator initiated discussion among the group on utilization patterns, preferences of providers and the barriers faced by clients when accessing MCH services. The presence of a moderator ensured the free flow of discussion and facilitated the exploration of emergent topics to obtain a more detailed insight. Another trained researcher accompanied the moderator and recorded noteworthy verbal and non-verbal gestures. Debriefing occurred at the end of each FGD when the moderator summarized key points from the discussion and sought group confirmation of data accuracy. Necessary clarification was sought where disagreement arose. All FGDs were tape recorded and transcripts were prepared based on audio recordings as well as notes taken during the discussions. The data were collected in Sindhi language. It was translated and transcribed by a researcher fluent in both Sindhi and English language. The responses were coded on free nodes using NVIVO software (Version 8.0). These nodes were then organized into parent (tree), child and sibling nodes. Parent node corresponds to each type of MCH service, sibling nodes contained information on: utilization of MCH service, preference for type of healthcare provider, and broader category of barriers, while child nodes denoted issues/reasons identified under each category. In addition, transcripts were read iteratively for deeper understanding of meaning. Interpretation and assertions about findings were made by keeping in view of the local context and cultural practices.

## 4. Findings

### 4.1 Utilization of MCH Services, Provider Preference and Barriers

Mothers and fathers reported low utilization of MCH services particularly of facility based births, post natal care (PNC), newborn checkup, immunization and family planning. However, most clients reported using antenatal care (ANC) during pregnancy; and facility based care for maternal complications and child illnesses. Those who used services, commonly preferred private clinics over public sector facilities. Participants highlighted a number of barriers to MCH service utilization including: lack of awareness about importance of service utilization, lack of functional public sector services, transportation costs, absence of female staff in health facilities, unaffordability of medicines in private sector, and low mobility of women due to security and cultural issues. Specific barriers for each type of service are listed in Table 2.

**Table 2:** Perceived barriers to utilization of MCH services

Maternal care			
**ANC**	**Facility based delivery**	**PNC**	**Maternal complications**
•Lack of awareness about importance of seeking ANC	•Lack of awareness about importance of facility based birth	•Lack of awareness about importance of seeking PNC	•Unaffordability of medicines and transport
•Long distance	•Unaffordability of medicines and transport		•Lack of female staff and medicines in public facilities
•Inadequate transport	•Fear of Caesarian Section and vaginal examination		•Poorly functional public sector facilities
•Lack of female staff in public facilities	•Difficult physical access		•Inadequate transport
•Poorly functional public sector facilities	•Lack of female staff in public facilities		
•Unaffordability	•Poorly functional public sector facilities		
•Limited mobility of women and security concerns	•Limited mobility of women and security concerns		
•Cultural Issues		

**Child care**			

**Newborn Care**	**Acute Illnesses**	**Immunization**	**Family Planning**
•Lack of awareness about importance of seeking newborn care	•Unaffordability of medicines and transport	•Lack of awareness about availability and schedule of vaccination	•Lack of awareness about availability of family planning services
	•Difficult physical access	•Fear of side effects	•Low effectiveness of contraceptives and fear of side effects
	•Poorly functional public facilities		•Irregular supply to existing users
			•Desire for a male child

#### 4.1.1 Antenatal Care

Most respondents did not consider it necessary to seek routine ANC in the absence of an ailment. However, those who were interested in seeking antenatal care were not satisfied with the services provided at public sector facilities, forcing clients to seek expensive private sector services. Expense of medicines and transport, long distance from private sector facilities, and inadequate transport facility were frequently cited barriers by the majority who were interested to seek care. Law and order issues in more remotely located villages and gendered norms also restricted the mobility of women who had to wait for escort from spouses and male relatives. One of the participants narrated,

“We do not consider it necessary to seek care without any ailment” (a father from Laoowari village, district Ghotki)

Another participant mentioned,

*“Poverty is the leading barrier. Also there is neither a lady doctor nor good quality medicines available in RHC. Staff attitude is also not good with patients and that is why people do not prefer to visit the RHC” (a father from Shahdadkot, District Qambar)*.

#### 4.1.2 Delivery Care

There were low levels of awareness on the importance of facility based delivery and most participants across all communities preferred home based delivery. Deliveries at the health facilities took place only in cases of complication such as obstructed labor or when traditional birth attendant (TBA) was not competent enough to handle the case.

*“When I was pregnant, my husband decided for institutional delivery. But I did not agree, I had overwhelmingly shown my wish to be delivered at home. I delivered first baby at private hospital (where) I suffered a lot of pains…… Later on when I delivered baby at home it was much better experience than that of the hospital” (A mother from village Rajukhanani, district Badin)*.

Commonly cited barriers to women seeking facility based births were long distance to facility, and difficulty in finding transport. Other barriers included the fear of Caesarian Section and vaginal examination. Shortage of female provider, blood bank and supplies at public sector facilities was a deterrent to use facility based services and most respondents were apprehensive of the expense of private sector facilities. Necessity of male consent for accessing hospital was required, particularly given the remote rural locations of villages where travel and safety of women was risky.

*“Almost all women deliver at home………We don’t have other low cost options. If Dai (TBA) feels that delivery at home is not possible then she refers the mother to deliver at a civil (Government) or private hospital*” *(A mother from village Aadabio, district Tharparkar)*.

#### 4.1.3 Maternal Complications

It was customary not to seek postnatal check-up unless there was a complication or threat to the mother’s/baby’s life. However, the importance of seeking facility based care in cases of maternal complication, such as excessive bleeding; vomiting and abnormal vaginal discharge was well recognized. In such cases, most of the mothers reported seeking treatment early from the nearest female doctor, Lady Health Visitor (LHV) or other private provider.

*“We get treatment in maternal complication because of fear of death or any complication or of miscarriage” (A mother from Naudero, district Larkana)*.

The better off families sought care from private sector facilities as they perceived these to be of good quality while poorest people were unable to approach formal healthcare services due to expense of both transport and private medical care. Non-availability of round the clock services and emergency care at nearby hospitals and inadequate transport network delayed use of services.

#### 4.1.4 Family Planning

Respondents were generally lukewarm about using contraceptives, but there were few refusals to use contraceptives. Low awareness about available contraceptive services was a major factor for underlying low utilization of contraceptives. There were also complaints of side effects of contraceptive pills and injections, low effectiveness, and irregular supply by health workers.

*“Some women of this area used injection but after getting this, they had started vaginal bleeding [due to which] we are scared of using contraceptives” (A mother from Humayoon Sarif, district Shikarpur)*.

#### 4.1.5 Immunization

The acceptance and demand for Polio vaccination at the community level was higher than the past due to ongoing polio campaigns and awareness messages through mass media. However they were not aware of other routine vaccinations. Few clients agreed to take vaccines at home but they were reluctant to approach a healthcare facility. Other reported barriers included: lack of information about the availability and schedule of immunization; and fear of side-effects.

*“They (Vaccinators/LHWs) come only for polio drops not for other vaccines even we don’t receive any education about benefits of vaccine” (A mother of village Akheraj, district Umarkot)*.

#### 4.1.6 Childhood Acute Illnesses

There was low awareness about the importance of routine newborn care and examination. However, there was high recognition of acute child illnesses and utilization of private health sector facilities was reported by most of the respondents. The care seeking for acute child illnesses depends upon multiple factors including unaffordable transport and medicine, perceived severity of illness, and distance from the healthcare facility. The well-off families receive care from private sector irrespective to severity of the illness and distance from health care facility, while the poor and those living in peripheral areas rely on home remedies. Mostly clients reported their mistrust of public health systems for care of child illnesses due to poor quality of care, and un-availability of medicine. Others reported inadequate transport; and issues of safety and security as main barriers for care seeking. A father explains traditional healing methods:

*“We wrap Beatle leaves round the chest and back of the child as first line therapy. Mostly children get relieved by this therapy, which we have been practicing for generations” (A father from district Khnoth Jamshoro)*.

### 4.2 Differential Barriers to MCH Services Utilization by LHW Covered and Non-Covered Areas

The study also differentiated between the type of barriers encountered by catchments covered and not covered by LHWs. Awareness of facility based deliveries, PNC, and immunization was similarly low across both LHW covered andnon-covered areas.

More respondents from LHWs covered areas were aware of the importance of seeking ANC as compared to those residing in non-covered areas. However, the use of health provider for ANC visits was reported to be limited in both, the better aware LHW covered areas and the less aware non-covered areas. Expense of transport to health care facilities and recourse to purchase of medicines and diagnostics from private outlets were cited as the main hurdles. One of the LHWs stated:

*“Those who don’t get ANC…. the reason is poverty! While they (women) realize that ANC visit is necessary but they (women) use to say, from where do we arrange money for transportation and medicine?” (LHW from Daur, district Nawabshah)*.

Similarly, respondents from LHW covered areas were relatively aware about use of contraceptives than those in non-covered areas but complained of the irregular supply of condoms and pills provided by LHWs. Those residing in LHW non-covered areas were less aware, however outright refusal to use contraceptives was less noticeable. Those not interested in using contraceptives either expressed the desire of having a male child or fatalistically believed that the number of children is pre-determined by God. As one of the respondents mentioned,

*“Everything is at God’s will; twins have been born even after having (contraception) pills” (A Father from district Larkana)*.

Respondents across both LHW covered and non-covered areas were less aware of the importance of delivery by skilled attendants, post natal care checkup and newborn checkups. Convenience and ease were the prime factors cited for delivering at home through the help of traditional birth attendants, hence avoiding the hassle, expense and stay over at health care facilities. Postnatal care visit by mother and newborn checkups were relatively unfamiliar concepts for respondents.

There was strong buying in to access emergency care in case of maternal complications and newborn acute illness in both LHW covered and non-covered areas. However, respondents from both areas cited delayed care seeking mainly due to unavailability of functional healthcare facilities. LHW covered villages were more closely located to health facilities and respondents here complained mainly of expense of going to private sector due to poorly functional government care facilities. This often resulted in depletion of personal savings or borrowing from neighbors and relatives to seek emergency care. There was general mistrust of using government hospitals.

*“The people, who do not have a single rupee (penny) in hand, seek care from civil hospital (public sector hospital)”* (*A Father from village Aadabio, district Tharparkar)*.

For respondents in LHW non-covered villages the long distance to health facilities was an additional deterrent. Poorly functioning peripheral government facilities made the issue worse as patients had to seek care further away in the district headquarter hospital or crossover to an adjoining district. Both mothers and their spouses from LHW covered and non-covered areas had little knowledge about the importance of routine childhood immunizations, schedule of immunization and access points. Differential barriers to MCH services utilization by outreach coverage are summarized in Table 3.

**Table 3:** Differential barriers to MCH services utilization by outreach coverage

Services	Barriers	

	*LHWs covered area*	*LHWs non-covered area*
**Antenatal Care**	Expense, Distance	Low awareness
**Facility based delivery**	Low awareness, ease of home based	Low awareness, ease of home based
**Postnatal care**	Low awareness	Low awareness
**Maternal complications**	Expense	Distance, expense
**Newborn care**	Low awareness	Low awareness
**Newborn acute illness**	Expense	Distance, expense
**Immunization**	Low awareness	Low awareness
**Family planning**	Irregular supply of contraceptives	Low awareness

## 5. Discussion

Qualitative studies are increasingly being recognized as helpful in the identification of barriers faced by individuals and communities in accessing healthcare services. Their value lies in their ability to provide a multi-dimensional and contextual insights not provided by quantitative studies.

Pakistan is one of the countries where progress towards MDGs has fallen behind targets. This study is particularly focused on the context of remote disadvantaged districts so as to better inform the placement and utilization of health innovations. The study firstly compares barriers across a range of MCH services within the context of remote districts, and secondly explores whether the presence of community health workers helps reduce community perceived barriers.

The study found that continued policy emphasis in expanding the number of government health care centers is not likely to yield results in rural disadvantaged areas of Pakistan. Low community awareness, an under-performing community health worker program, governance issues related to proper functioning of health facilities, and lack of transportation networks are key barriers to health facility utilization for MCH services. These constraints particularly impact facility based births, pregnancy care visits, well-baby checkups and childhood immunization.

Community barriers differ across preventive and curative MCH services. Communities are willing to access preventive care services such as if these are provided at the villages, sidestepping the hassle of going to health care facilities, contrary to common perceptions. This is partially due to lukewarm efforts at demand generation within the community and also due to long distances to health facilities in these remote rural districts. Client refusal due to cultural and religious reasons is not a key barrier to use of preventive MCH services. In contrast, there is a strong demand for accessing health facilities in case of maternal complications and acute child illness. However, poorly functional health centers, difficult physical access, and expense of transportation fare and purchase of medicines deter families from timely access. Other qualitative studies have reported quality of care (Gebrehiwot, Goicolea, Edin, & Sebastian, 2012) and expense of health care (Spangler & Bloom, 2010) as important deterrent to use of health services.

There are important lessons to be learnt particularly within the context of remote rural districts. Barriers for preventive services are clearly different from those of curative services and require a two pronged approach. In the case of preventive services, task shifting from facility to the community level is required with development of cost effective and simpler packages of care. Moreover, better governance of preventive outreach programs may be beneficial. Child illness and maternal and newborn emergency care will require different modalities. Poor governance of LHWs has been reported by independent quantitative assessments of the program with only 40% of LHWs receiving feedback from their supervisors and 59% of supervisors visiting respective catchments (Oxford policy management, 2009). Studies show that community health workers can more successfully change certain community practices when supported by training in problem solving skills, supportive supervision, and having a supply of services responsive to community needs (Glenton et al., 2013; Lewin et al., 2010; Okuga, Kemigisa, Namutamba, Namazzi, & Waiswa, 2015). In the case of emergency obstetric and newborn services, better governance is required of existing secondary care centers rather than increases in the already large network of government health centers. Further, transportation issues are outstanding barriers and often beyond the scope of health ministries. Cross-sectoral collaboration with the local governments to provide transport networks, community transporter links, and safe law and order for mobility of women will be as important as investment in health services by health ministries.

While some of our findings confirm the existing global evidence, others are contextually different. The findings concur with maternal care studies from other settings showing clients perceiving childbirth as a natural process and skilled providers are only needed in the case of complication (Griffiths & Stephenson, 2001; Matsuoka, Aiga, Rasmey, Rathavy, & Okitsu, 2010; Mrisho et al., 2009; Nabukera, Batwala, Mulogo, Barry, & Salihu, 2006). However, family planning literature from the developing countries settings is increasingly showing a demand for services, and highlighting supply gaps as the major barrier (Mugisha & Reynolds, 2008). Additionally, this study shows that there is insufficient awareness amongst clients to seek MCH services from health facilities. Qualitative barrier studies on childhood immunization are few, and report fear and misconception leading to refusal in areas of low vaccination coverage (Benin, Wisler-Scher, Colson, Shapiro, & Holmboe, 2006). Our findings show clientele as not unwilling, but largely unaware and often under-covered by basic outreach services. Difficult physical access has been reported less by other studies, with staff attitudes at health facilities, decision making dynamics and costs of private consultations being more commonly reported (Afsana & Rashid, 2001; Gebrehiwot et al., 2012; Moyer et al., 2014; Tabatabaie, Zahra, & AbouAli, 2012).

The second thrust of the study was to look at differences in community barriers between LHW covered and non-covered areas. There is surprisingly low awareness for facility based delivery, PNC, newborn checkups and childhood immunization, across both LHW covered and non-covered areas. Better awareness is only seen for ANC services and family planning services and these also are confined to the LHW covered areas. This shows that there are critical areas of preventive care- related to the period around delivery and to childhood immunizations- that go unaddressed. It calls for better preparation of outreach workers to expand from ANC and family planning counseling to childhood immunization and promotive care around birth.

Our findings concur with quantitative evidence from the Pakistan Demographic and Health Survey (2013) that shows nearly two-thirds of women in rural areas (66.7%) are receiving antenatal care from skilled personnel, but comparable figures are much lower for birth related indicators with only 44.4% women being delivered by skilled personnel, 40.8% sought skilled PNC, and 34.4% had newborn checkup within 24 hours of childbirth. Routine immunization figures reported by PDHS (2013) are even lower with only 48.4% children aged 12-23 months had all basic vaccinations in rural Pakistan.

The strength of the study is in uncovering the perspective of disadvantaged rural communities, rather than that of the mainstream population. While most qualitative studies are confined to limited geographical areas, this study had the participation of a large number of districts. In addition, it provides a comparison of the types of barriers reported across a range of MCH services rather than solely focusing on a particular service. It also importantly highlights differences in the constraints experienced between catchments served and not served by community health workers. The limitations of this study lie in the exclusion of mother-in-laws who enjoy significant decision making power at a household level over maternity and child care. In addition, the study data collection was limited to a single method of data collection, rather than a triangulation of methods.

## 6. Conclusion

In the context of remote rural areas, the traditional emphasis on continued investment in health facility infrastructure is not likely to address poor MCH service utilization rates. There is a community demand to utilize health facilities for preventive services, but only if services are provided in villages. Preventive MCH services require a concerted effort to build community awareness, task shifting from facility to community for provision of services, and re-energization of CHWs programs. There is strong community demand to utilize health facilities for maternal and child emergencies, but its translation into timely access requires significant support for transport networks and fewer but more functional health care centers.
